# Epidemiology, Characteristics, and Outcomes of ICU-Managed Homeless Patients: A Population-Based Study

**DOI:** 10.1155/2018/3869652

**Published:** 2018-03-27

**Authors:** Lavi Oud

**Affiliations:** Division of Pulmonary and Critical Care Medicine, Department of Internal Medicine, Texas Tech University Health Sciences Center at the Permian Basin, Odessa, TX, USA

## Abstract

**Background:**

The population-level demand for critical care services among the homeless (H) remains unknown, with only sparse data on the characteristics and outcomes of those managed in the ICU.

**Methods:**

The Texas Inpatient Public Use Data File and annual federal reports were used to identify H hospitalizations and annual estimates of the H population between 2007 and 2014. The incidence of ICU admissions in the H population, the characteristics of ICU-managed H, and factors associated with their short-term mortality were examined.

**Results:**

Among 52,206 H hospitalizations 15,553 (29.8%) were admitted to ICU. The incidence of ICU admission among state H population rose between 2007 and 2014 from 28.0 to 96.6/1,000 (*p* < 0.0001), respectively. Adults aged ≥ 45 years and minorities accounted for 70.2% and 57.6%, respectively, of the growth in volume of ICU admissions. Short-term mortality was 3.2%, with odds of death increased with age, comorbidity burden, and number of failing organs.

**Conclusions:**

The demand for critical care services was increasingly high among the H and was contrasted by low short-term mortality among ICU admissions. These findings, coupled with the persistent health disparities among minority H, underscore the need to effectively address homelessness and reduce barriers to longitudinal appropriate prehospital care among the H.

## 1. Introduction

Homelessness remains an intractable problem in high-income countries [1]. Although challenges remain in accurately tracking homeless populations [1, 2], annual estimates show progressive decrease in the homeless population in the United States (US) [2]. The homeless have high frequency of comorbid conditions, often exceeding that in the nonhomeless population, and an especially high prevalence of mental illness, including common alcohol- and substance-related disorders [1]. With increasingly recognized ageing of the homeless population in the US [3], it has been shown that age-related comorbid conditions tend to develop at an earlier age among the homeless [1].

The prevalent comorbidities and homelessness itself are combined with possibly inadequate control of chronic illness and lack of timely interventions for otherwise manageable acute illness, related to barriers in accessing medical care [4, 5]. These factors likely underlie the high utilization of inpatient healthcare resources by the homeless, with high hospitalization and readmission rates and with higher inpatient costs, substantially exceeding those among their nonhomeless counterparts [6, 7]. Acute health crises among the homeless can, in turn, progress to critical illness requiring care in the ICU.

Data on the epidemiology of contemporary demand for critical care services, as well as the characteristics and outcomes of homeless patients requiring critical care, can inform healthcare policy and healthcare system-level efforts to mitigate preventable progression to critical illness and reduce healthcare costs and would further inform clinicians' decision-making.

However, data on critically ill homeless patients remain sparse. Two recent studies from France [8] and Canada [9] described high severity of illness, resource utilization, and hospital mortality of ICU-managed homeless patients. However, these studies were limited by small cohort size and examination of single-center data. There have not been, to our knowledge, reports on the demand for critical care services in the homeless population.

Our study objectives were to examine, at a population-level, the evolving demand for critical care services among the homeless and the characteristics, resource utilization, and outcomes among those admitted to ICU. We studied the homeless population in Texas, a state with a large, demographically diverse population, with a high-quality administrative data that systematically reports on admission to ICU. Because there has been a progressive and substantial decline of estimated homeless population in Texas (−28% between 2007 and 2014 [2]), we hypothesized that there will be corresponding decrease in the demand for critical care services in this population in the state.

## 2. Materials and Methods

This was a retrospective, population-based cohort study. The study was determined to be exempt from formal review by the Texas Tech Health Sciences Center's Institutional Review Board due to use of a publicly available, deidentified dataset. Parts of the methods described below were previously reported [10].

### 2.1. Data Sources and Study Population

The Texas Inpatient Public Use Data File (TIPUDF) has been used to identify the study population. The use of TIPUDF has been previously described [11]. In brief, TIPUDF is an administrative dataset maintained by the Texas Department of State Health Services [12] and includes deidentified inpatient discharge data on the demographic, clinical, resource utilization, and outcome domains from state-licensed, nonfederal hospitals and captures 93% to 97% of all hospital discharges in the state. The state of Texas masks gender data of hospitalizations with a diagnosis of infection with the human immunodeficiency virus (HIV), ethanol, or drug abuse. In addition, the state provides only broad age group data for hospitalizations with a diagnosis of infection with HIV infection, or with alcohol or drug abuse, to protect patient privacy. Thus, age groups for the latter category of hospitalizations are reported in the dataset as 0–17 years, 18–44 years, 45–64 years, 65–74 years, and ≥75 years. Because TIPUDF provides discharge-level, rather than patient-level information, precluding accounting for repeated admissions in the dataset, we report number of hospitalizations and ICU admissions as units of analysis, rather than number of patients.

We identified hospitalizations during the years 2007–2014 who were homeless, using the* International Classification of Diseases, Ninth Revision, Clinical Modification* (ICD-9-CM) code V60.0. Hospitalizations with ICU admission served as the primary analytic cohort. Data on the homeless population in Texas was obtained from the annual estimates reported by US Department of Housing and Urban Development [2, 13]. The reports do not include state-specific age categories or race/ethnicity. We used the US Census Bureau data for censal and intercensal estimates of Texas population and its age and race/ethnicity strata [14].

### 2.2. ICU Admissions

Identification of hospitalizations with ICU admission was based on unit-specific revenue codes for an intensive care unit or a coronary care unit. Intermediate care units or step-down units were not included. Because administrative datasets do not include information of the temporal course of hospitalization, it could not be determined whether ICU admissions occurred at the start of hospitalization or later during hospital course. Similarly, the proximate indications for ICU admission were not available.

### 2.3. Outcomes

The primary outcome measure was short-term mortality of ICU-admitted hospitalizations, defined as the combination of hospital mortality and discharge to hospice. Secondary outcomes included temporal patterns of ICU utilization and the burden of chronic illness, organ failure, and resource utilization.

### 2.4. Study Covariates

We abstracted the following covariates of ICU admissions. (a) The first covariate is demographics (age, gender, race/ethnicity [categorized as non-Hispanic black (black), non-Hispanic white (white), Hispanic, and other], and health insurance [categorized as Private, Medicare, Medicaid, Uninsured, and other]). (b) The second is comorbid conditions, based primarily on the Deyo modification of the Charlson Comorbidity Index [15, 16]; in addition, we collected data on mental illness, based on the Clinical Classification Software (CCS) developed by the Healthcare Cost and Utilization Project [17]. The CCS group of mental illness, based on specific ICD-9-CM codes, includes among other subcategories alcohol- and substance-related disorders. We further abstracted diagnoses of depression and alcohol- and substance-related disorders because they represent the most common subcategories of mental illness reported in the homeless population [1] (see Supplementary Table 1); finally, we collected data on hospitalizations with diagnoses of malnutrition [18], obesity, and tobacco use (see Supplementary Table 1). (c) The third covariate is medical or surgical hospitalization (based on the primary diagnosis-related grouping). (d) The fourth is organ failures [19]. (e) The fifth is use of invasive mechanical ventilation (termed mechanical ventilation hereafter), hemodialysis, and blood transfusion (see Supplementary Table 1). (f) The sixth is hospital length of stay. (g) The seventh is total hospital charges. (h) The eighth is teaching designation of the hospital. (i) The ninth is hospital disposition [categorized as routine home, home with home health, transfer to another hospital, transfer to a nursing facility, death, hospice, and a leave against medical advice]. (j) Finally, the tenth is year of hospitalization.

### 2.5. Data Analysis

Data on categorical variables was summarized as numbers and percentages, while continuous variables were reported as mean (standard deviation [SD]) or median (interquartile [IQR]). The *t*-test, the Mann-Whitney test, and Kruskal-Wallis test were used for comparison of continuous variables, as appropriate. The Cochran-Armitage test for trend was used for categorical variables and the Jonckheere-Terpstra trend test was used with Kruskal-Wallis comparisons.

We examined the demand for critical care services as incidence of ICU admission among the homeless population in Texas, the rate of ICU admission among all hospitalized homeless patients, and the rate of ICU admission across age strata of homeless hospitalizations. Because state-specific data on age and race/ethnicity are not provided in reports on the homeless population in the US [2] and in order to further explore the evolving demand for critical care services among the homeless in context of the growing Texas population and changing state demographics, we examined the incidence of ICU admission of homeless patients with the general state population as denominator. Additional stratified analyses of the incidence of ICU admission of the homeless among the general population in the state were carried out across age and race/ethnicity categories. The annual incidence of ICU utilization among the homeless population in Texas is reported as number of ICU admissions per 1,000 population and that indexed for the general population in the state is described as the number of ICU admissions per 100,000 population.

Temporal trends of the incidence of ICU admissions among the homeless population and the general population in Texas were examined using least squares regression of log-transformed data. Similar approach was employed to evaluate the annual changes in the number of failing organs among ICU admissions. Because ICU admissions among those aged < 18 years were rare, we excluded this subgroup from the age-stratified temporal trend analyses. Trends were reported as average annual percent change (AAPC) and corresponding 95% confidence intervals (95% CI). Annual changes in the rates of ICU admissions among hospitalizations of homeless patients were assessed by logistic regression.

Because temporal changes in ICU utilization may be affected by changes in threshold of ICU admission, rather than changing needs, we examined the corresponding temporal trends of measures of severity of illness, using the number of organ failures. We chose the number of failing organs as a measure of severity of illness because administrative data do not provide the clinical information commonly used to determine traditional severity of illness scores. The number of failing organs is well-documented as predictor of mortality in the critically ill [20, 21] and demonstrated good discrimination in our cohort, with C-statistic 0.87.

Total hospital charges were adjusted for inflation using the consumer price index and reported as 2014 US dollars [22]. TIPUDF and the state of Texas do not provide tools for conversion of hospital charges to costs.

We used multivariate logistic regression modeling to examine predictors of short-term mortality of ICU admissions, using a purposeful selection approach for selecting modeled covariates [23]. As a first step, univariate logistic regressions were carried out, with covariates with *p* < 0.2 considered for multivariate analysis. Candidate covariates were then examined for multicollinearity. An initial multivariate logistic model was then performed using backward stepwise selection method. Next, we added back, one at a time, all the covariates not selected for entry in the original multivariate model to those retained in the backward selection approach. This last step allowed identification of covariates that were not significantly related by themselves to short-term mortality among ICU admissions but made important contribution in the presence of other covariates. The final multivariate model included the following covariates: age, race/ethnicity, health insurance, Deyo comorbidity index, transfer from another hospital, admission to a teaching hospital, number of failing organs, malnutrition, mental illness, blood transfusion, mechanical ventilation, and year of hospitalization. Because no short-term mortality was reported among ICU admissions aged < 18 years we excluded this group from modeled predictors of short-term mortality among ICU admissions. In addition, because gender was reported only in a minority of ICU admissions due the high frequency of alcohol- and substance-related disorders, we examined the association of gender with short-term mortality as an exploratory analysis, using the multistep purposeful covariate selection approach described earlier. We reported model findings as adjusted odds ratios (aOR) and their corresponding 95% CI.

Data management was performed using Excel and Access (Microsoft, Redmond, Washington) and statistical analyses were performed with MedCalc version 17.5.5 (MedCalc Software, Ostend, Belgium). A 2-sided *p* value < 0.05 was considered statistically significant.

## 3. Results

### 3.1. Patterns of ICU Utilization

**Figure 1:**
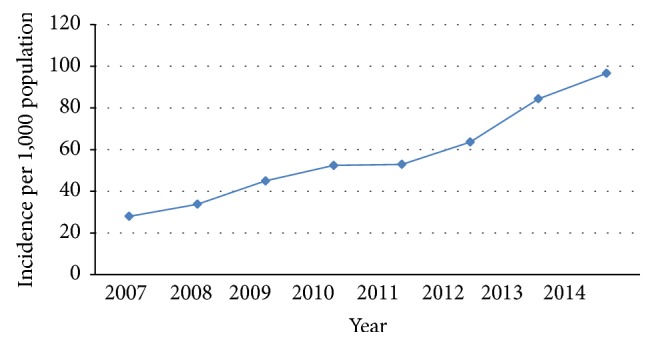
Incidence of ICU admissions among the homeless population in Texas.

We identified 52,206 hospitalizations of homeless patients, of which 15,553 (29.8%) were admitted to ICU. The annual incidence of ICU admission during study period among the homeless population in Texas is outlined in Figure 1. The annual total and age- and race/ethnicity-stratified incidence of ICU admission homeless among the general state population are detailed in Table 1.

The incidence of ICU admission among the homeless population rose between 2007 and 2014 from 28.0 to 96.6 per 1,000 population, respectively (AAPC [95% CI] 17.0 [14.3–19.7]; *p* < 0.0001). The incidence of ICU admissions of the homeless out of the general population in Texas increased for the whole cohort (rising by 115% between 2007 and 2014) and for all examined age- and race/ethnicity strata, with the highest change rate noted among Hispanic ICU admissions (rising by 229% between 2007 and 2014).

The growth in the volume of ICU admissions between 2007 and 2014 (increasing from 1,114 to 2,752, resp.) was predominantly by those aged ≥ 45 years, who accounted for 70.2% of the change, and by minority homeless, accounting for 57.6% of the change.

The rate of ICU admissions among hospitalized homeless patients changed relatively little over time, though it was statistically significant (odds ratio [95% CI] 1.071 [1.008–1.025]; *p* = 0.0001). The rate of ICU admission among homeless hospitalizations rose progressively with age, increasing from 14.7% among those aged < 18 years to 37.1% among those 65 years or older (*p* < 0.0001 for trend).

### 3.2. The Characteristics, Resource Utilization, and Outcomes of ICU Admissions

The characteristics of ICU admissions are detailed in Table 2. ICU admissions involved rarely those aged < 18 years (0.2%) and those aged ≥ 65 years were uncommon (6.2%). Gender was reported in 5,467 (35.2%) ICU admissions, with males accounting for 74%. Private health insurance was uncommon (12.7%), while 45.1% had no health insurance. One or more comorbid conditions tracked under the Deyo comorbidity index were reported in 65.8% of ICU admissions, though the number of these comorbidities was low (mean [SD] 1.2 [1.4]), as was the Deyo comorbidity index. Mental illness was reported in 13,050 (83.9%) ICU admissions, with depression and alcohol- and/or substance-related disorders accounting for 88.8% of these ICU admissions. Only 439 (2.8%) ICU admissions of the homeless did not have at least one comorbidity tracked by the Deyo comorbidity index and/or mental illness. One or more organ failures were reported in 6,472 (41.6%) ICU admissions. The mean number of failing organs rose over time, increasing between 2007 and 2014 from 0.53 to 0.78, respectively (AAPC [95% CI] 5.5 [2.6–8.4]; *p* = 0.0037). The median (IQR) Deyo comorbidity index scores and the number of failing organs, while low in all groups, increased across the < 18-year, 18–44-year, 45–64-year, and ≥65-year age strata (0 [0-0], 0 [0-1], 1 [0–3], and 2 [0–3]; *p* < 0.0001 and *p* < 0.0001 for trend; 0 [0-1], 0 [0-1], 0 [0-1], and 1 [0-1]; *p* < 0.0001 and *p* < 0.0001 for trend, resp.).

Hospital length of stay did not change significantly between 2007 and 2014 (*p* = 0.1348), while median [IQR] total hospital charges rose by 48% ($22,469 [13,894–42,856] versus $33,266 [20,516–60,478], resp.; *p* < 0.0001). When examined as a proportion of hospital days and hospital charges among all hospitalizations of the homeless, the total hospital days and hospital charges among ICU admissions accounted for 33.8% and 47.5%, respectively.

Discharge to another facility was reported in 14.2% of ICU admissions. The short-term mortality was 3.2%. Hospital mortality preceded by ICU admission accounted for 83.8% of all hospital deaths among hospitalized homeless patients.

### 3.3. Predictors of Short-Term Mortality

The association of examined covariates with short-term mortality on univariate analysis is outlined in Table 3, and the corresponding findings on multivariate logistic regression are detailed in Table 4.

Older age, increasing burden of comorbidities under the Deyo comorbidity index, and malnutrition were associated with increased odds of short-term mortality, as were increasing number of organ failures and need for life-support interventions. When compared with private health insurance, both Medicaid and lack of health insurance were associated with increased short-term mortality. On the other hand, ICU admissions with reported mental illness had markedly lower odds of death, and homeless blacks admitted to ICU tended to have lower odds of short-term mortality. On exploratory analysis on the prognostic role of gender among ICU-managed admissions of homeless patients without diagnoses of HIV infection, alcohol abuse, or drug abuse, women tended to have lower odds of short-term mortality (aOR [95% CI] 0.605 [0.359–1.022]; 0.0604) [see Supplementary Table 2].

## 4. Discussion

In this population-based cohort study, the key findings included high and rising demand for critical care services among the homeless population in Texas, with high rate of admission to ICU, once hospitalized. The growing demand for critical care services was driven predominantly by older and minority homeless. Homeless patients admitted to ICU had near-universal presence of one or more comorbid conditions, including high rate of mental illness. ICU admissions of the homeless accounted for a disproportionately high fraction of hospital days and total hospital charges of all homeless hospitalizations. Short-term mortality was low among ICU admissions and was adversely affected by type of health insurance, burden of comorbid conditions, and severity of illness, while mental illness and care at teaching facilities appeared protective.

We report for the first time, to our knowledge, on the population-level demand for critical care services among the homeless, which by 2014 reached in Texas nearly 1 ICU admission per 10 homeless people per year. In addition, the rate of the rising demand for critical care services by the homeless in Texas outpaced the corresponding growth rate of the general population in the state. These findings were unexpected, given the reported 28% decline in the estimated homeless population in Texas between 2007 and 2014 [2]. Increasing ICU utilization can occur due to reduced threshold for ICU admission. However, this explanation is unlikely, given the rising severity of illness of those admitted to ICU during study period.

Our findings can be placed in perspective by comparison with the reported utilization of critical care services by the general population in the US. In a preliminary report by Milbrandt and colleagues, the estimated incidence of ICU admission in 2005 was 28 per 1,000 population [24], essentially identical to our findings for the year 2007. However, ICU admissions in the general population include disproportionately high number of patients aged 65 years or older, who represent half of ICU admissions in the US [25], reflecting their much higher burden of chronic illness. On the other hand, only a small minority of ICU admissions in our cohort was in the latter age group, suggesting a disproportionately higher than expected demand for critical care services among the homeless, given their overall younger age. Although more recent data were not reported, to our knowledge, on ICU utilization in the general population in the US, it seems implausible that it grew over 3-fold, as was noted among the homeless population in Texas.

The rising rate of ICU utilization by the homeless was driven predominantly by older adults, reflecting in turn the ageing homeless population and the demonstrated increasing burden of chronic comorbidities and severity of illness with rising age. Although whites accounted for half of ICU admission, most of the growing demand for critical care services was accounted by minority ICU admissions, underscoring the persistent and growing health disparities even within the homeless population. The highest rate of growth in ICU admissions occurred among Hispanic patients, reflecting their predominance in population growth due to external migration to Texas [26]. The observed temporal patterns of ICU utilization suggest that unless there is a substantial change in addressing the development and persistence of homelessness, the demand for critical care services among the homeless is expected to continue its rapid escalation.

The overall high level of demand for critical care services may also reflect the prevalent mental illness among the homeless population. Prior reports demonstrated poorer level of control of chronic illness among patients with mental illness [27]. In addition, findings of a recent study by Wunsch and colleagues suggested that mental illness may have a role in increasing the risk of critical illness [28]. The later hypothesized risk may be magnified under conditions of homelessness. Finally, the well-documented high rates of hospital [29] and ICU [8] readmissions among the homeless, being markedly higher than that in the nonhomeless population, have contributed to the high ICU utilization among the former.

Our findings extend earlier reports of markedly high rate of hospitalization among the homeless, as compared with the general population, to ICU admission rates once patients are hospitalized. Nearly 1 in 3 hospitalizations of homeless patients included admission to ICU, as compared with 18% [30] to 22% [24] among hospitalizations in the general population in the US. The sources of the marked difference are unclear. However, it may be hypothesized that the difference may stem from higher complexities of care of acute health crises among the homeless due to common barriers to timely and ongoing outpatient care, which may allow manageable acute illness to deteriorate more than among their nonhomeless counterparts [1]. In addition, as noted earlier, the highly prevalent mental illness in the homeless population may have further contributed to the high rate of ICU utilization, once hospitalized.

Several sociodemographic characteristics of the present cohort contrasted prior reports about the homeless population. Although black patients admitted to ICU were overrepresented, as compared to their fraction in the general population, similar to prior studies [31, 32], homeless Hispanic ICU admissions occurred at a markedly lower rate than expected for Texas population (22% versus 40%, resp., in 2014 [14]). The latter findings were unexpected, given that Hispanics in the state represent the largest population group living in poverty [33], and Hispanics were previously reported to be slightly overrepresented among hospitalizations of homeless patients [32]. However, in a recent study from New York, Frencher at al. reported that Hispanics were underrepresented among homeless hospitalizations, as compared with those with low socioeconomic status [34]. The sources for the conflicting findings are unclear.

Nearly half of ICU admissions of homeless patients lacked health insurance, nearly double the rate reported for homeless hospitalizations in the US [32]. Our findings reflect the overall high rate of the uninsured in Texas, being the highest in the US [35], which likely contributed to the high demand for critical care services, similar to reports in the general population [36].

Although we found near universal presence of one or more of the examined comorbid conditions among homeless ICU admissions, the overall number of comorbidities among individual ICU admissions was low, which may have contributed to their low short-term mortality (see below). There have not been, to our knowledge, comparable examination of comorbidity burden in other hospitalized homeless populations.

Details of the type and number of organ failures have not been reported in other studies of homeless ICU admissions [8, 9] or contemporary population-level studies of ICU admissions in the general US population, precluding direct comparisons of severity, as traditional severity of illness scores could not be derived from administrative data. However, recent reports of ICU admissions of the homeless described patients with much higher severity of illness than in our cohort, as judged by the higher use of mechanical ventilation (44% [8] and 56% [9]) and high mortality (20.8% [8] and 29% [9]). The difference likely represents a combination of both local practice patterns at study hospitals and lower availability of ICU beds outside the US [37]. On the other hand, organ failure occurred in 22.1% of ICU admissions in the general population in the US [24], representing nearly half the rate observed in our cohort, though use of mechanical ventilation among ICU admissions of homeless patients in our cohort was similar to that reported for ICU admissions in the general US population (10.0 versus 9.5%, resp.) [24]. However, neither the number of failing organs nor their severity was reported for ICU admissions in the general population [24], precluding, as noted earlier, direct severity comparison with our study (see further comment below).

The impact of ICU utilization on the overall hospital resource utilization among hospitalized homeless patients has not been previously reported, to our knowledge. Our findings extend prior reports which documented high healthcare resource utilization by homeless patients, as compared with their nonhomeless counterparts [7]. Thus, ICU admissions in the general population in the US accounted for markedly smaller fraction of hospital days (20.2%) and total hospital costs (30.5%) among all hospitalizations, as compared to our cohort [24].

Hospital mortality among ICU admissions of homeless patients in our cohort was, as noted earlier, markedly lower than in previously reported cohorts of homeless ICU patients [8, 9] and was lower than that of ICU admissions in the general US population (8.8%) [38]. Prior reports did not include discharges to hospice, which are increasingly prevalent in the US [39]. The single center-based cohorts used for prior reports on homeless ICU admissions may limit the generalization of their outcome data, while the higher mortality in these studies likely reflects the noted higher acuity of ICU admissions in countries with lower availability of ICU beds than the US [40]. The lower hospital mortality among ICU admissions of homeless patients in the present study, as compared to those in the general population in the US, likely represents both markedly younger age and lower overall burden of chronic illness. In addition, although, as noted earlier, the overall rate of organ failure was higher among ICU-managed homeless patients in the present cohort than among ICU admissions in the general US population [24], it may be hypothesized that the lower mortality rate among the former could have reflected a lower number and severity of involved organ failures in affected patients. Further studies are needed to examine the validity of the latter hypothesis at a population level.

The fraction of hospital deaths preceded by admission to ICU among hospitalizations of homeless patients substantially exceeds that for hospitalization in the general population in the US (83.8% versus 58.9% [41], resp.). The observed differences may have resulted in part from the documented higher level of preference for life support interventions among the homeless as comparted to patients with chronic illness [42] and, importantly, may have been related to a recently documented markedly lower availability of substitute decision-makers on admission and lower frequency of family involvement in end-of-life decisions among critically ill homeless patients, as compared with the nonhomeless [9]. Our findings and those reported by Smith et al. [9] underscore the urgent need to explore community and hospital level approaches to assure that both care and care setting consistently meet the goals of care of critically ill homeless patients.

The population-level contrast between the high and rising demand for critical care services among the homeless and their likely lower severity of illness and short-term mortality, as compared to ICU-managed patients in the general population in the US, underscore the need to effectively address the burden of homelessness, prehospital care barriers, and more efficient use of critical care resources through community-care, policy-care, and healthcare system-level efforts.

Our findings on the adverse prognostic impact of older age and lack of health insurance on short-term mortality of homeless patients admitted to ICU contrast those reported by Bigé et al. [8]. The sources of the difference may be related in part to differences in case-mix and the healthcare system between the US and France and to a smaller cohort size managed at a single ICU. Our results extend to the homeless population findings of prior studies documenting excess short-term mortality among uninsured critically ill patients in the general population in the US [36]. On the other hand the findings of near-doubling of the odds of death among ICU admissions of homeless patients with Medicaid insurance contrast lack of adverse prognostic impact in the general ICU population [36]. It is unclear whether the observed differences about the adverse prognostic impact Medicaid insurance are state-specific or apply across the US.

Only sparse data were reported to date on the impact of mental illness on short-term mortality of ICU patients, showing mixed findings, with either increased risk [43], no effect [44], or lower risk of death [45]. The association between mental illness and short-term mortality has not been reported, to our knowledge, among hospitalized homeless patients. Our findings of markedly lower odds of short-term mortality among ICU admissions of homeless patients with mental illness were unexpected, given the aforementioned poorer control of other comorbidities, possible increased risk of critical illness, and increased all-cause long-term mortality associated with the latter [46]. Further studies are needed to examine the prognostic impact of mental illness in the critically ill.

Finally, the lack of change in adjusted short-term mortality over time in our cohort, despite corresponding ageing and increasing severity of illness, suggests possible care improvements over time.

Our findings should be considered in the context of several important limitations, related to the retrospective study design and use of administrative data. First, hospitalizations of the homeless may have been improperly identified and possibly underestimated, in part as the ICD-9-CM code V60.0 is not a billable code. However, homeless patients were identified using similar approach in prior epidemiological studies [47, 48]. Second, challenges to accurately quantify the homeless population may lead to its underestimation. However, identical methodology was used for annual population estimates across states [2]. Thus, the accuracy of annual estimates of the homeless population would be unlikely to affect substantially temporal trends. Third, as noted earlier, the timing and indications for ICU admission were not available, nor were data on the clinical variables that have affected clinicians' triage to ICU. Fourth, although we examined a large cohort of ICU admissions, we cannot exclude residual confounding in our predictive modeling due to the observational, retrospective study design. Finally, although we studied a large heterogeneous population, our findings may not reflect the populations of ICU-managed homeless patients in the remainder of the US or internationally.

## 5. Conclusions

In this first population-based study in the US, we demonstrated an increasingly high demand for critical care resources in the homeless population, driven predominantly by older adults and minorities. ICU admissions of the homeless accounted for a disproportionately high fraction of all hospital days and hospital charges among hospitalized homeless patients. Socioeconomic factors and especially inadequate health insurance, along with the burden of chronic illness and severity of illness, have adversely affected short-term mortality, while mental illness appeared to have a protective impact. However, despite the high demand for critical care services and substantial use of hospital resources, exceeding the corresponding features for ICU admissions in the general population in the US, homeless patients admitted to ICU had low short-term mortality, as compared to the latter. These contrasting results highlight the need for additional studies in other healthcare settings to both corroborate our findings and help guide future efforts to improve healthcare and especially reduce the burden of ICU-requiring care among the homeless.

## Figures and Tables

**Table 1:** Incidence of ICU admissions of homeless patients in Texas^a^.

Group	2007	2008	2009	2010	2011	2012	2013	2014	AAPC (95% CI)^b^	*p* value
All	4.7	5.6	6.7	7.3	7.6	8.4	9.4	10.1	10.4 (8.5–12.4)	<0.0001
Age (years)										
18–44	4.1	4.6	5.1	6.4	5.8	7	7.6	8.4	9.9 (7.6–12.2)	<0.0001
45–64	12.3	14.6	18.2	18.2	20.5	20.8	24.3	26.3	10.0 (7.6–12.4)	<0.0001
≥65	1.9	3.6	3.6	4.2	4	5.8	6.1	5.9	14.5 (7.8–21.1)	0.0018
Race/ethnicity										
white	4.9	5.9	7.7	8.6	9.1	9	11.7	11	11.4 (7.6–15.1)	0.0003
Hispanic	1.7	1.9	2.4	2.8	3	4.5	4.4	5.6	17.4 (14.7–20.1)	<0.0001
Black	12.8	16.5	17.2	18	17.4	14.6	20.2	24.9	6.1 (0.6–11.5)	0.0333
Other	4.9	4.6	5.3	5.1	6.8	16.5	4.7	5.3	5.1 (−11.5–21.7)	0.4818

^a^Texas state population used as denominator, reported as number of ICU admissions per 100,000 population;  ^b^AAPC: average annual percent change; 95% CI: 95% confidence interval.

**Table 2:** The characteristics, hospital resource utilization, and outcomes of ICU admissions.

Variables	ICU admissions
	*n* = 15,553
Age, years (%)	
<18	33 (0.2)
18–44	4,954 (31.9)
45–64	9,565 (61.7)
≥65	971 (6.2)
Gender (n = 5,467 [%])^a^	
Female	1,430 (26.2)
Race/ethnicity (%)	
White	7,876 (50.6.0)
Hispanic	2,649 (17.0)
Black	4,242 (27.3)
Other	786 (5.1)
Health insurance (%)	
Private	1,976 (12.7)
Medicare	2,587 (16.3)
Medicaid	3,493 (22.5)
Uninsured	7,018 (45.1)
Other	479 (3.1)
Missing	54 (0.3)
Deyo comorbidity index (mean [SD])	1.6 (2.1)
Comorbidities (%)	
Lung disease^b^	3,726 (24.0)
Diabetes^b^	3,327 (21.4)
Liver disease^b^	2,894 (18.6)
Renal disease^b^	1,517 (9.8)
Congestive heart failure^b^	2,291 (14.7)
Malignancy^b^	504 (3.2)
HIV^b^	466 (3.0)
Mental illness	13,050 (83.9)
Depression	5,395 (34.7)
Drug-related disorders	5,794 (37.3)
Alcohol-related disorders	6,537 (42.0)
Smoking	8,648 (55.6)
Malnutrition	1,517 (9.8)
Obesity	1,037 (6.7)
Transfer from another hospital	655 (4.2)
Type of hospital admission (%)	
Medical	13,669) (87.9)
Surgical	1,884 (12.1)
Teaching hospital	9,072 (58.3)
Number of organ failures (%)	
0	9,081 (58.4)
1	4,001 (25.7)
2	1,564 (10.1)
3+	907 (5.6)
Type of organ failures (%)	
Respiratory	2,116 (13.6)
Cardiovascular	981 (6.3)
Renal	2,975 (19.1)
Hepatic	389 (2.5)
Hematological	1,546 (9.9)
Metabolic	1,156 (7.4)
Neurological	1,125 (7.2)
Mechanical ventilation (%)	1,565 (10.0)
Hemodialysis (%)	510 (3.3)
Blood transfusion (%)	1,452 (9.3)
Hospital length of stay (days)	
Mean (SD)	7.4 (10.6)
Median (IQR)	5 (3–8)
Total hospital charges^c^	
Mean (SD)	50,234 (66,383)
Median (IQR)	29,494 (18,109–54,418)
Hospital disposition (%)	
Routine home	11,351 (73.0)
Home with home health	186 (1.2)
Another hospital	1,306 (8.4)
Nursing facility	908 (5.8)
Leave against medical advice	1,116 (7.2)
Death	383 (2.5)
Hospice	119 (0.8)
Missing	184 (1.2)

^a^Gender was masked in 10,086 ICU admissions;  ^b^part of the Deyo comorbidity index;  ^c^adjusted for inflation to 2014 US dollars.

**Table 3:** Univariate logistic regression of predictors of short-term mortality among ICU admissions.

Variables	Odds ratio (95% CI)	*p* value
Age		
18–44 years	1	
45–64 years	2.291 (1.805–2.908)	<0.0001
≥65 years	2.913 (2.026–4.189)	<0.0001
Gender		
Male	1	
Female	0.390 (0.246–0.618)	0.0001
Race/ethnicity		
White	1	
Hispanic	1.372 (1.092–1.723)	0.0065
Black	0.779 (0.617–0.982)	0.0353
Other	1.664 (1.181–2.344)	0.0036
Health insurance		
Private	1	
Medicare	1.154 (0.781–1.704)	0.4703
Medicaid	2.050 (1.455–2.890)	<0.0001
Uninsured	1.505 (1.082–2.093)	0.0151
Other	1.348 (0.731–2.485)	0.3379
Deyo comorbidity index	1.417 (1.375–1.461)	<0.0001
Mental illness	0.511 (0.417–0.625)	<0.0001
Depression	0.2331 (0.176–0.307)	<0.0001
Substance-related disorders	0.572 (0.467–0.700)	<0.0001
Alcohol-related disorders	1.290 (1.080–1.542)	0.005
Smoking	0.745 (0.624–0.890)	0.0012
Malnutrition	4.908 (4.040–5.963)	<0.0001
Obesity	0.916 (0.633–1.325)	0.6442
Transfer from another hospital	1.589 (1.100–2.293)	0.0134
Weekend admission	0.964 (0.788–1.179)	0.7238
Type of hospital admission		
Medical	1	
Surgical	1.081 (0.817–1.430)	0.5843
Teaching hospital	0.912 (0.762–1.091)	0.3151
Number of organ failures	3.130 (2.291–3.354)	<0.0001
Mechanical ventilation	19.519 (16.125–23.627)	<0.0001
Hemodialysis	3.849 (2.859–5.182)	<0.0001
*Blood transfusion*	6.041 (4.989–7.314)	<0.0001

**Table 4:** Multivariate logistic regression of predictors of short-term mortality among ICU admissions.

Variables	Adjusted odds ratio (95% CI)	*p* value
Age		<0.0001
18–44 years	1	
45–64 years	1.190 (0.900–1.574)	0.2201
≥65 years	1.536 (1.038–2.273)	0.0316
Race/ethnicity		
White	1	
Hispanic	1.152 (0.869–1.528)	0.3265
Black	0.796 (0.612–1.035)	0.0898
Other	1.202 (0.781–1.849)	0.4020
Health insurance		
Private	1	
Medicare	0.813 (0.505–1.308)	0.3950
Medicaid	1.981 (1.464–2.682)	<0.0001
Uninsured	1.502 (1.141–1.978)	0.0037
Other	1.025 (0.497–2.115)	0.9442
Deyo comorbidity index^a^	1.338 (1.286–1.391)	<0.0001
Mental illness	0.557 (0.434–0.716)	<0.0001
Malnutrition	2.177 (1.714–2.766)	<0.0001
Transfer from another hospital	2.221 (1.399–3.527)	0.0007
Teaching hospital	0.695 (0.559–0.864)	0.0011
Number of organ failures^b^	1.979 (1.810–2.165)	<0.0001
Mechanical ventilation	6.116 (4.728–7.913)	<0.0001
Blood transfusion	1.726 (1.355–2.199)	<0.0001

^a^per 1 point;  ^b^per organ failure.

## Supplementary Materials

Supplementary Materials Supplementary Table  1: ICD-9 codes for comorbidities and procedures. International Classification of Diseases, Ninth Revision, Clinical Modification (ICD-9-CM) codes used to identify selected comorbidities and procedures. Supplementary Table  2: multivariate analysis of predictors of short-term mortality among ICU admissions with reported gender. Multivariate logistic regression of predictors of short-term mortality among ICU admissions with reported gender.
