# Supplementary material for: Epidemiology, Characteristics, and Outcomes of ICU-Managed Homeless Patients: A Population-Based Study
Source: Biomed Res Int. 2018 Mar 27;2018:3869652. doi: 10.1155/2018/3869652 (PMC5892254; doi:10.1155/2018/3869652)
Supplement: Supplementary Materials — Supplementary Table 1: ICD-9 codes for comorbidities and procedures. International Classification of Diseases, Ninth Revision, Clinical Modification (ICD-9-CM) codes used to identify selected comorbidities and procedures. Supplementary Table 2: multivariate analysis of predictors of short-term mortality among ICU admissions with reported gender. Multivariate logistic regression of predictors of short-term mortality among ICU admissions with reported gender. [file 3869652.f1.docx]

Online Data Supplement to:

**Epidemiology, Characteristics, and Outcomes of ICU-Managed Homeless Patients: a Population-Based Study**

Lavi Oud, MD

**Supplementary Table 1. International Classification of Diseases*,* Ninth Revision*,* Clinical Modification (ICD-9-CM) codes used to identify selected comorbidities and procedures.** (Where only 3 or 4-digit codes are listed, all associated subcodes are included)

**Variable ICD-9-CM codes**

Depression 293.83, 296.2, 296.3, 296.4, 296.5, 296.6, 296.7, 296.8, 296.9, 300.4, 311

Alcohol-realted disroders 291.XX, 303.XX, 305.0X, 357.5, 425.5, 535.3, 535.30, 535.31, 571.0, 575.1, 575.2, 575.3, 760.71, 980.0

Substance-realted disroders 292.XX, 304.XX, 305.2X, 305.3X, 305.4X, 305.5X, 305.6X, 305.7X, 305.8X, 305.9X, 648.30, 648.31, 648.32, 648.33, 648.34, 655.50, 655.51, 655.53, 760.72, 670.73, 760.75, 779.5, 965.00, 965.01, 965.01, 965.09, V6542

Obesity 278.00, 278.01, 278.03, 649.1, 649.10-649.14, V85.30-V85.45

Tobacco use 305.1X, V15.82

Mechanical ventilation 96.70-96.72

Hemodialysis 38.95, 39.95

Blood transfusion 99.00-99.09, V58.2

|  | | | | | | | | | | | |
| --- | --- | --- | --- | --- | --- | --- | --- | --- | --- | --- | --- |
|  |  |  |  |  |  |  |  |  |  |  |  |

**Supplementary Table 2. Multivariate logistic regression of predictors of short-term mortality among ICU admissions with reported gender**

| **Variables** | | | **Odds ratio (95% CI)** | | | **p value** |
| --- | --- | --- | --- | --- | --- | --- |
| **Gender** |  |  |  |  |  |  |
| Male |  | 1 | | | |  |
| Female |  | 0.605 (0.359-1.022) | | | | 0.060 |
| **Race/ethnicity** |  |  |  |  |  |  |
| White |  | 1 | | | |  |
| Hispanic |  | 1.025 (0.620-1.696) | | | | 0.921 |
| Black |  | 0.681 (0.438-1.058) | | | | 0.088 |
| Other |  | 1.321 (0.710-2.455) | | | | 0.378 |
| **Health insurance** |  |  |  |  |  |  |
| Private |  | 1 | | | |  |
| Medicare |  | 1.204 (0.514-2.818) | | | | 0.668 |
| Medicaid |  | 1.946 (1.198-3.162) | | | | 0.0071 |
| Uninsured |  | 1.636 (1.046-2.561) | | | | 0.031 |
| Other |  | 0.987 (0.271-3.594) | | | | 0.985 |
| **Deyo comorbidity index** |  | 1.282 (1.196-1.374) | | | | <0.0001 |
| **Mental illness** |  | 0.497 (0.335-0.735) | | | | 0.0005 |
| **Smoking** |  | 0.727 (0.498-1.061) | | | | 0.0987 |
| **Malnutrition** |  | 1.782 (1.143-2.776) | | | | 0.0106 |
| **Transfer from another hospital** | | 3.507 (1.647-7.468) | | | | 0.0011 |
| **Teaching hospital** |  | 1.012 (0.689-1.486) | | | | 0.950 |
| **Number of organ failures** |  | 1.971 (1.685-2.305) | | | | <0.0001 |
| **Mechanical ventilation** |  | 6.698 (4.302-10.427) | | | | <0.0001 |
